# Dietary patterns and breast cancer risk, prognosis, and quality of life: A systematic review

**DOI:** 10.3389/fnut.2022.1057057

**Published:** 2023-01-20

**Authors:** Yuan Bu, Junchao Qu, Siqi Ji, Jingxin Zhou, Mengxin Xue, Jiling Qu, Huiping Sun, Yongbing Liu

**Affiliations:** ^1^School of Nursing and Public Health, Yangzhou University, Yangzhou, Jiangsu, China; ^2^Jiangsu Vocational College of Medicine, Yancheng, Jiangsu, China

**Keywords:** dietary patterns, breast cancer, breast cancer risk, prognosis, quality of life

## Abstract

**Background:**

Statistics indicate that the morbidity of breast cancer is increasing globally, and its (overall figures) incidence has now surpassed that of lung cancer for the first time. The relation between a whole dietary pattern, rather than of a single food or nutrient, and breast cancer (BC) should be examined for findings to capture the complexities of diet and the potential for synergism between dietary components. Hence, the effects of dietary patterns on breast cancer have recently attracted increasing attention.

**Objective:**

To systematically review the effects of dietary patterns on breast cancer risk, prognosis, and quality of life in survivors.

**Methods:**

This systematic review was conducted following PRISMA guidelines and was registered in PROSPERO. Data from Ovid, China Biomedical Literature Database, Wanfang Data Knowledge Service Platform, CNKI, PubMed, Weipu, The Cochrane Library, Duxiu Data, ProQuest, Embase, Web of Science, and Scopus Database were retrieved and evaluated.

**Results:**

A total of 47 studies that investigated the association between eating patterns and breast cancer were identified. Ten studies evaluated the effect of the model on treatment outcome and prognosis of breast cancer and two cross-sectional studies examined the influence of dietary patterns on quality of life. The resulting favorable dietary patterns were shown to regulate metabolic biomarkers, antioxidants, anti-inflammatory agents, and protective genes, and inhibit cell proliferation and invasion.

**Conclusion:**

Numerous studies have examined the effects of healthy eating, plant-based, anti-inflammation, low-fat, and other favorable dietary patterns in relation to breast cancer. However, few studies reported significant associations and the studies had limitations, suggesting that the current findings should be interpreted with caution.

**Systematic review registration:**

https://www.crd.york.ac.uk/prospero/, CRD4202 2350171.

## Introduction

The most recent estimates of the International Agency for Research on Cancer (IARC) indicated that breast cancer (BC) was the most prevalent cancer in women worldwide with 2.3 million diagnoses in 2020, thus surpassing lung cancer for the first time. BC is responsible for approximately 685,000 deaths per year, and it is the fifth leading cause of cancer-related deaths in women (1).

A previous study of the link between the gut and mammary glands found that diet could alter the gut microbiome and breast tumor microenvironment, thereby influencing tumorigenesis (2). Current research suggests that nutritional status affects cell invasion and lipid metabolism in BC (especially triple-negative breast cancer) (3), and can thus impact BC risk, treatment outcomes, and quality of life in survivors. Dietary research has shifted from studying single nutrients or foods to holistic dietary patterns (4), given that analysis of single nutrients and foods cannot address the effects of interactions between or changes in multiple nutrients and food components ingested together. In nutritional epidemiology, nutrients present in food are expressed based on their biological significance, and a new concept of food synergy has been established. The most reliable evidence for a link between diet and health outcomes is thus obtained by determining the overall effects of different eating patterns, considering the mutual effects of their nutrients (5, 6).

Breast cancer (BC) is the focus of extensive research, especially in countries with a high rate of the disease. Levels of consumption of animal products and all types of drinks are nearly twice as high and the consumption of plant- and grain-based foods is lower in countries with a high rate of BC. For example, in Mediterranean countries, where animal products are consumed at twice the rate of plant-based foods, the morbidity rate of BC is 51/100,000 (7). These findings highlight the need to explore the impact of dietary patterns on BC. However, most patients do not have sufficient understanding of the effects of dietary patterns and clinical factors on BC risk, disease outcomes, and quality of life in survivors, and a lack of understanding of relevant dietary patterns may lead to patients being diagnosed with advanced disease of BC (8).

A Mediterranean-style diet has been shown to reduce the risk of BC (9), while a low-fat diet reduced mortality in post-menopausal patients (10), and healthy eating patterns improved the quality of life of patients with BC (11). However, the role of dietary patterns in populations with specific BCs is inconclusive. We therefore systematically analyzed the effects of the components of different dietary patterns on BC, and determined which characteristics of the population were most affected by specific dietary patterns.

## Materials and methods

### Search process

The International System Review Registry Platform (PROSPERO) registration number for this project is CRD42022350171. The study is presented according to PRISMA guidelines for systematic reviews. Ovid, China Biomedical Literature Database, Wanfang Data Knowledge Service Platform, CNKI, PubMed, Weipu, The Cochrane Library, Duxiu Data, ProQuest, Embase, Web of Science, and the Scopus database were searched for relevant literature on BC and dietary patterns, using subject words and free words. The reference lists of the identified studies were also searched for additional studies. Dandamudi et al. published a systematic review of studies published up to January 2017 (12). The current search time was limited to studies published between 01 January 2017 and 30 July 2022, with no language restrictions.

The search identified articles with the following terms in the title or abstract: “Breast Neoplasms” OR “Breast Neoplasm” OR “Neoplasm, Breast” OR “Breast Tumors” OR “Breast Tumor” OR “Tumor, Breast” OR “Tumors, Breast” OR “Neoplasms, Breast” OR “Breast Cancer” OR “Cancer, Breast” OR “Mammary Cancer” OR “Cancer, Mammary” OR “Cancers, Mammary” OR “Mammary Cancers” OR “Malignant Neoplasm of Breast” OR “Breast Malignant Neoplasm” OR “Breast Malignant Neoplasms” OR “Malignant Tumor of Breast” OR “Breast Malignant Tumor” OR “Breast Malignant Tumors” OR “Cancer of Breast” OR “Cancer of the Breast” OR “Mammary Carcinoma, Human” OR “Carcinoma, Human Mammary” OR “Carcinomas, Human Mammary” OR “Human Mammary Carcinomas” OR “Mammary Carcinomas, Human” OR “Human Mammary Carcinoma” OR “Mammary Neoplasms, Human” OR “Human Mammary Neoplasm” OR “Human Mammary Neoplasms” OR “Neoplasm, Human Mammary” OR “Neoplasms, Human Mammary” OR “Mammary Neoplasm, Human” OR “Breast Carcinoma” OR “Breast Carcinomas” OR “Carcinoma, Breast” OR “Carcinomas, Breast” AND “Dietary pattern.”

### Eligibility criteria and study selection

The inclusion criteria were: (1) cohort study, randomized controlled trial (RCT), cross-sectional research, or case-control study; (2) full text provided; and (3) study evaluated the effects of eating patterns or dietary interventions on BC risk, all-cause/specific mortality, recurrence, and quality of life. The exclusion criteria were: (1) dietary studies combined with physical activity; (2) studies without full text, results, and key data; (3) studies of any population not explicitly defined as cancer survivors; (4) cell and animal experiments, conference abstracts without full text, reviews, and meta-analyses; and (5) duplicate studies or several publications from the same study.

### Data fetch and quality evaluation

Articles were identified and the following data were retrieved by two researchers: general patient information, sample size, assessment of eating patterns, indicators of disease change, outcomes (relationship between dietary patterns and BC, 95% confidence intervals, odds ratios, correlation coefficients, hazard ratio, and p-values), and identification of confounding variables associated with BC (e.g., sex, smoking, tumor classification, estrogen, TNM staging, education, menarche, age, and menopausal age). The quality of case-control and cohort studies was assessed by the Newcastle-Ottawa scale (NOS), which includes selection of study population, comparability between groups, and outcome/exposure. The total score ranges from 0 to 9, with a score ≥ 6 indicating high-quality. Details of the NOS scale are provided in document S1. RCTs were assessed using Cochrane risk bias maps, with each aspect receiving a low, high, or unclear rating. The quality of the cross-sectional studies was based on the Joanna Briggs Institute (JBI) quality evaluation. This was a descriptive systematic review.

## Results

**FIGURE 1:**
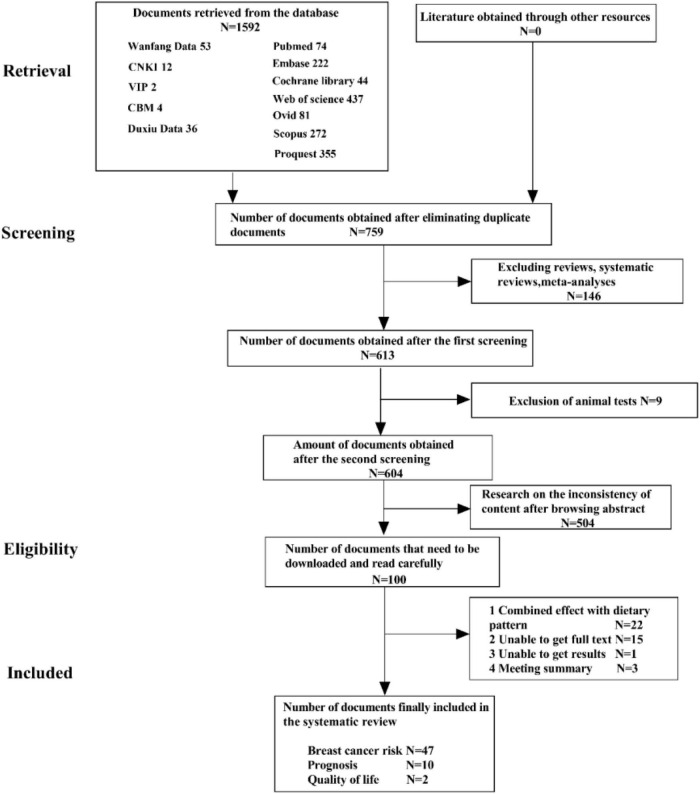
Document screening flow chart.

The PRISMA flowchart is shown in Figure 1. The preliminary search identified 1592 articles, of which 759 articles remained after excluding duplicate studies, and 107 articles remained after excluding systematic reviews, meta-analyses, animal experiments, and content discrepancies. The full texts of these articles were read, and the selected research findings, specific data, and comparator patterns are shown in Table 1. Forty-seven studies assessed the positive and negative effects of dietary patterns on cancer risk, 10 assessed the impact of eating patterns on treatment outcomes and prognosis, and two assessed the effects of eating patterns on quality of life in patients after a cancer diagnosis. This review included 35 case-control studies, 19 cohort studies, two cross-sectional studies, and three RCTs. The results of quality evaluations of the studies are presented in Tables 2–4 and Supplementary Figure 2. All previously conducted studies, except for three RCTs with a high risk of bias, were of high quality. In the three RCTs, random sequences were generated by using a permuted block algorithm and simple randomization, respectively. For allocation hiding, only Chlebowski et al. (13) described implementation points with hidden methods, while other two did not. It is difficult to blind the participants and researchers in dietary studies, and the three RCTs were therefore not blinded. However, the outcome evaluators were blinded in two of the studies (13, 14), but not in the third study (15). The mean and standard deviation were used to estimate the missing data in all three papers, and the reasonable effect size of the missing data did not affect the final observation results. There was no risk of selective reporting bias or other bias in any of the studies.

**TABLE 1:** Dietary patterns: Disease risk, prognosis, and survival quality.

Authors, country	Study	Sample size	Dietary assessment	Dietary pattern	Findings	Covariates included in the model
Castelló et al. (Spain) (37)	A case-control study	1063 BCs and 1469 healthy controls (20–85y), patients with recently diagnosed (< 6 month)	FFQ (154 items); 1 y prior	Western, Prudent, and Mediterranean dietary patterns	Western pattern increases the venture of BC (OR:1.53, 95%CI1.15–2.02) Mediterranean diet high adherence reduces BC risk (OR:0.72, 95%CI:0.53–0.98)	Pausimenia status, time of life, BMI, family history of BC, PA, smoking, caloric intake, alcohol intake, province, education, time at the first delivery
Sadeghi et al. (Iran) (16)	A case-control study	145 BCs and 149 controls	SQFFQ (168 items) 1 y prior	Unhealthy and healthy dietary patterns	Participants who had higher scores for unhealthy dietary patterns and healthy dietary patterns were more (OR: 4.12, 95%CI: 2.31–7.3) and less (OR: 0.17, 95%CI: 0.30–0.95) prone to develop breast cancer, respectively. A healthy dietary pattern reduced the risk of HER-2 positive breast cancer (OR: 0.11, 95% CI: 0.04–0.25).	Age, marriage, education, smoking, menarche, contraceptives, number of deliveries, menopausal status, ethnic history of breast, supplements, HRT
Lu et al. (China) (62)	A case-control study	818 BCs and 935 Controls BCs with recently diagnosed (< 12 month)	FFQ (23 items); 1 y prior	salty, vegetarian, sweet and traditional Chinese	The traditional Chinese pattern was associated with a lower risk of BC among both pre- and post-menopausal women (4th vs. 1st quartile: OR for pre-and post-menopausal women was 0.47 and 0.68, respectively). Women with high factor scores of the sweet pattern also showed a decreased risk of BC (4th vs. 1st quartile: OR for pre- and post-menopausal women was 0.47 and 0.68, respectively).	Residence, BMI, age, education, household income (5 y prior), menopausal status. age at menarche, abortion, WHR, age at first live birth
Guinter et al. (US) (63)	A nested case-control study	393 controls and 260 BCs	DQX (137items); 1 y prior	estrogen-related dietary pattern (ERDP)	A 1-unit increase in the ERDP score was associated with total (HR:1.09, 95%CI:1.01–1.18), invasive (HR:1.13; 95%CI:1.04–1.24) and ER positive (HR:1.13, 95%CI:1.02–1.24) BC risk.	PHT use at baseline, BMI, alcohol, parity, vigorous, PA, Family history of BC
Krusinska et al. (Poland) (22)	A nested case-control study	82 controls and 47 cases (40–79y)	FFQ-6(62 items); 1 y prior	Non-Healthy, Prudent, Margarine, Sweetened Dairy	The risk of BC risk was three-times higher (OR: 2.90; 95% Cl: 1.62–5.21; *p* < 0.001) in the upper tertile of the “Non-Healthy” pattern (reference: bottom tertile)	Family history, number of children, age, BMI, socioeconomic, abuse of alcohol, PA, menarche, menopausal, contraceptive, HRT, smoking, vitamin/mineral supplement, molecular of BC subtypes
Krusinska et al. (Poland) (23)	A nested case-control study	140 controls and 140 BCs (40–75 y)	FFQ-6 (62 items);	Dressings, Non-healthy, Sweet low-fat dairy, Prudent	The risk of BC was lower in the average (3–5 points) and high (6–8 points) levels of the “Polish-aMED” score compared to the low (0–2 points) level by 51% (OR: 0.49; 95%Cl: 0.30–0.80; *p* < 0.01; adjusted) and 63% (OR: 0.37; 95% Cl: 0.21–0.64; *p* < 0.001; adjusted), respectively. In the middle and upper tertiles compared to the bottom tertile of the “Prudent” DP, the risk of cancer was lower by 38–43% (crude) but was not significant after adjustment for confounders. In the upper compared to the bottom tertile of the “Non-healthy” DP, the risk of cancer was higher by 65% (OR: 1.65; 95% Cl: 1.05–2.59; *p* < 0.05; adjusted).	Age, sex, type of cancer, BMI, SES, PA, smoking, alcohol abuse.
Jalali et al. (Iran) (40)	A case-control study	134 BCs and 267 hospitalized controls	FFQ (168 items); 1 y prior	Dietary Inflammatory Index	DII pattern enhanced the risk (OR quartile 4 vs. 11/4 2.64, 95% CI: 1.12–6.25; Ptrend1/4 0.01), particularly premenopausal population (OR quartile 4 vs. 11/4 5.51, 95%CI: 1.45–20.93; Ptrend1/4 0.005);	Waist, WHR, Age, Weight, Height, BMI, Hip, Menarche age, Marriage age, First pregnancy age, Childbirth number, Child number, Breastfeeding time, OCP use time, PA, Daily energy intake, Miscarriage history, HRT, Benign breast diseases history, BC family history, Cancer family history, Night bra use, Day bra use, Inflammatory disease history, NSAIDs, Menopausal status, Marital status, Educational level, Smoking, Vitamin D
Heidari et al. (Iran)(18)	A case-control study	134 BCs and 267 controls	FFQ (168 items);	healthy and unhealthy	Unhealthy eating patterns enhance the risk (OR: 2.21; 95%CI: 1.04-4.69; Ptrend = 0.009).	first live birth, Age, Weight, Height, BMI, WHR, Total energy intake, PA, Menarche age, Marriage age, Breastfeeding duration, Menopausal status, Pre-Postmenopause, Smoking, Education Family history of cancer, Day bra use, Night bra use, Vitamin D supplements, OCP use
Shridhar et al. (Indian) (52)	A case-control study	400 BCs and 354 controls (30–69 y) BCs with recently diagnosed(< 6mo)	FFQ (184 items); 1 y prior	lacto-vegetarians (Only dairy products are eaten in animal products), lacto-ovo-vegetarians (included eggs), non-vegetarians	Breast cancer risk was lower in lacto-ovo-vegetarians compared to both non-vegetarians and lacto-vegetarians with OR (95% CI) of 0.6 (0.3–0.9) and 0.4 (0.3–0.7), respectively.	Breastfeeding, Age, Education, Age at menarche in years, Religion, Diabetes, Hypertension, Family history of cancer, BMI, Waist circumference, Age during first pregnancy (in years), months/child, Menopause, Age at menopause in years, Years of estrogen exposure. Oral contraceptive pills, PA, Moderate-vigorous,
Sedaghat et al. (Iran) (20)	A case-control study	267 hospitalized controls and 134 newly diagnosed BCs	FFQ (168 items); 1 y prior	Healthy Eating Index 2010 (HEI-2010)	higher HEI-2010 scores were associated with lower risk of BC only among premenopausal women (multivariate adjusted OR1/4 0.27, 95%CI: 0.10–0.69; P for trend 1/40.02).	Height, BMI, Waist, Hip, WHR, Age, Menarche age, Marriage age, First pregnancy age, Child number, Breastfeeding time, OCP use time, PA, Daily energy intake, Abortion history, HRT, BC family history, Cancer family history, Inflammatory disease history, Anti-inflammatory drugs, Menopausal status, pre-menopause, post-menopause, Marital status, University education, smoking Vitamin D supplement
Turati et al. (Italy, Switzerland) (36)	A case-control study	3034 BCs and 3392 controls	FFQ (78 items); weekly intake	Mediterranean Diet	Compared to a MDS of 0–3, the ORs for BC were 0.86 (95% CI, 0.76–0.98) for a MDS of 4–5 and 0.82 (95% CI, 0.71–0.95) for a MDS of 6–9 (p for trend = 0.008).	Oral contraceptive, Age, education, BMI, PA, smoking, parity, menopausal status/age, HRT history of diabetes, family history (first-degree relatives), non-alcohol energy intake, alcohol
Marzbani et al. (Iran) (19)	A case-control study	212 BCs and 408 controls	The middle-aged integrated health care form (11items);	Unhealthy dietary patterns	The OR of BC associated with consumption of less vegetables was 2.8 (OR: 2.8; 95%CI:1.7–4.5). The OR of BC in women who consumed more soft drinks (OR: 2.8; 95%CI:1.9–4.3), industrially produced juices (OR:2.7; 95%CI: 1.1–6.5) and solid oils (OR:1.9; 95% CI:1.3–3.0). The OR of BC in more fried foods was 4.5 (95%CI: 2.1–9.4). The OR of BC in excessive sweets was 2.6 (95%CI:1.7–3.9).	Age, Education, Location, Marital status, Job, Insurance coverage, BMI
Heidari et al. (Iran) (58)	A case-control study	136 BCs and 272 hospital-based controls (d ≥ 25 y)	FFQ (168 items); 1 y prior	DASH: Günther’s, Dixon’s, Fung’s, and Mellen’s	Women in the highest categories of the Mellen’s and Günther’s scores had lower odds of BC than those in the lowest quintiles (Mellen’s OR:0.50; 95%CI:0.62–0.97; Ptrend:0.02; Günther’s OR:0.48; 95%CI:0.25–0.93; P-trend:0.05). BC risk was reduced in postmenopausal women with higher scores on Mellen’s index (OR:0.24; 95%CI:0.08–0.68; Ptrend:0.04).	Supplement, age, BMI, first live birth, PA, smoking, total energy, vitamin D, family history
Stasiewicz et al. (Poland) (25)	A case-control study	230 controls, 190 primaries (21 d prior before recruitment) BCs (40.0–79.9y)	FFQ-6 (62 items); 1 y prior	Pro and Neutral-inflammatory	The lower OR of BC was associated with the higher adherence to the “Pro-healthy/Neutral-inflammatory” profile (OR:0.38; 95%Cl:0.18–0.80; *p* < 0.01 for the higher level vs. lower level, crude model; OR for one-point score increment:0.61; 95%Cl: 0.42–0.87; *p* < 0.01, adjusted model). The higher OR of BC was associated with the higher adherence to the “Unhealthy/Pro inflammatory” profile (OR:3.07; 95%Cl: 1.27–7.44; *p* < 0.05 for the higher level vs. lower level, adjusted model; OR for one-point score increment:1.18; 95%Cl: 1.02–1.36; *p* < 0.05, adjusted model).	Age, BMI, socioeconomic, PA, smoking, alcohol, age at menarche, times of full-term pregnancies, contraceptive, HRT, family history (first- or second-degree relative), vitamin/mineral, molecular subtypes of BC
Hajji-Louati et al. (France) (48)	A case-control study	872 BCs and 966 controls (25-75y)	FFQ (153 items); 1 y prior	Dietary Inflammatory Index (DII)	The OR contrasting quartile 4 to quartile 1 was 1.31 (95%CI: 1.00–1.73; p-trend = 0.02).	Menopausal, BBD, Age, menarche, department, first full-term pregnancy, family history (first-degree relatives), parity, breastfeeding, menopausal hormone therapy, BMI, socioeconomic, oral contraceptive, education
Sasanfar et al. (Iran) (49)	A case-control study	412 BCs( ≤ 1 year) and 456 healthy controls.	FFQ (168 items); 1 y prior	Unhealthful plant-based diet index (uPDI); healthful plant-based diet index (hPDI); Plant-based diet index (PDI)	a greater score of hPDI was inversely associated to the risk of breast cancer (OR: 0.63; 95%CI:0.43–0.93, *P* = 0.01), pre- and postmenopausal women in the highest quartile of hPDI score had lower risk of BC than those in the lowest quartile.	Age, energy, PA, family history, educational level, parity, marital status, BMI
Aghamohammadi et al. (Iran) (60)	A case-control study	350 BCs and 700 controls. (≥ 30y)	DS-FFQ (106 items); daily intake	MIND	women in the top tertile of the MIND diet score had 60% lower odds of BC than women in the bottom tertile (OR:0.40, 95%CI: 0.29–0.55).	Smoking, Age, breastfeeding, energy intake, BMI, menopausal status, education, SES, residency, family history, PA, marital status, alcohol, supplement use,
Soltani et al. (Iran) (57)	A case-control study	350 BCs and 700 controls(≥ 30y) (< 6 mo)	DS-FFQ (106 items); daily intake	DASH	individuals in the highest quartile of the DASH diet score had 85% lower odds of BC than women in the bottom quartile (OR: 0.15; 95% CIs: 0.09–0.24).	Marital status, Age, alcohol, BMI, supplement, PA, energy intake, menopausal status, education, residency, family history of BC, smoking, breastfeeding
Cao et al. (China) (34)	A case-control study	818 BCs and 935 healthy controls	FFQ (149 items);	Prudent, Chinese traditional, Western, Picky	Prudent-factor was associated with a lower risk of breast cancer [4th vs. 1st quartile: OR:0.70, 95%CI:0.52–0.95], Picky-factor/class was associated with a higher risk (4th vs. 1st quartile: OR:1.35, 95% CI:1.00–1.81).	First full-term delivery, Age, BMI, history of benign disease, area, education, smoking, moderate PA, OCP, HRT, menarche, parity, family history, breastfeeding, height, energy intake, menopausal status
Cao et al. (China)(80)	A case-control study	695 BCs, 804 controls	FFQ (149 items);	Prudent, Chinese traditional, Western, Picky	Compared with Prudent class, Picky class was associated with a higher risk (OR:1.42, 95%CI = 1.06–1.90)	Age (diagnosis/enrollment/menopausal/first full term delivery/menarche), BMI, area, education, smoking, tea, alcohol, PA, oral contraceptives, HRT, history of benign disease, parity, breastfeeding, height, energy intake, family history
Cao et al. (China) (35)	A case-control study	818 BCs, 935 controls	FFQ (149 items);	Mediterranean dietary pattern (MDP)	High adherence (highest quartile) to the MDP decreased the risk of breast cancer among post- but not premenopausal women, respectively (OR:0.54, 95% CI:0.38–0.78 and 0.90, 0.53–1.53).	Education, Age (diagnosis, enrollment, menarche, first full term delivery), area, smoking, family history, PA, oral contraceptives, HRT, history of benign disease, parity, breastfeeding, BMI,
Hammad et al. (Jordan) (45)	A case-control study	200 BCs and 200 controls (≤ 3 mo) (≥ 20 y)	FFQ (109items); 1 y prior	Dietary inflammatory index (DII)	Stratified analyses by obesity status showed that overweight/obese participants in the highest DII tertile had a >75% increased BC risk (OR: 1.77 95%CI: 1.01–3.12) compared with participants in the lowest tertile.	Education, BMI, number of pregnancies, Contraceptive use, Age, energy, lactation, cigarette, family history
Jacobs et al. (South African) (56)	A case-control study	396 BCs and 396 controls	QFFQ 1 m prior	Pattern1: Manufactured food; Pattern2: Traditional; Pattern3: Cereal-dairy breakfast	Pattern (2,3) bring down the risk (highest tertile versus lowest tertile, OR:0.72, 95%CI: 0.57–0.89, p-trend = 0.004 and OR:0.73, 95%CI: 0.59–0.90, p-trend = 0.004, respectively)	Breast-feeding, Age, ethnicity, exogenous hormones, individual income, level of education, smoking, height, waist circumference, habitual PA, age (first pregnancy, menarche, menopause), full-term pregnancy, time since menopause, parity, duration of exclusive breastfeeding, HRT, alcohol, HIV positivity, miss-reporting of energy, total energy intake, family history
Flores-Garcia et al. (Mexico) (24)	A case-control study	509 BCs and 509 controls	FFQ (119items); 1 y prior	Pattern 1: cereals, meat, high fat, and sugary), Pattern 2: corn, legumes, vegetables)	The first pattern was positively associated with BC (OR 1/4 12.62; 95%CI: 7.42-21.45); the Second pattern was inversely associated with BC (OR 1/4 0.50; 95%CI: 0.40-0.62).	Energy, Alcohol, BMI, estrogen, education, smoking, family history
Sheikhhossein et al. (Iran) (81)	A case-control study	150 BCs and 150 controls	FFQ (147items); Daily intake	MIND	we found no significant association between the MIND diet score and odds of BC, either before (ORs for comparing T3 vs. T1: 0.818; 95%CI:0.469-1.42, P-trend = 0.48) or after controlling for potential confounders (ORs for T3 vs. T1:1.32; 95%CI: 0.31-5.64, P-trend = 0.633).	Age, energy intake, age at first pregnancy, PA, education, marital status, menopause status, socioeconomic, alcohol, smoking, vitamin, medical history, OCP, age at first menarche, menopause, weight (18y), number of children, length of breastfeeding, family history, iron, folate, BMI
Rigi et al. (Iran) (50)	A case-control study	350 BCs and 700 controls	FFQ (106items); 1 y prior	Plant-based dietary patterns: PDI, hPDI, uPDI	Individuals in the highest quartile of PDI had 67% lower odds of BC than those in the lowest quartile (OR: 0.33; 95%CI: 0.22–0.50). Individuals with the greatest adherence to hPDI were 36% less likely to have BC than those with the lowest adherence, in the fully adjusted model (OR:0.64; 95%CI 0.43–0.94). In terms of uPDI, women in the top quartile had a 2.23 times greater chance of BC than those in the bottom quartile (OR:2.23; 95%CI:1.48–3.36).	Marital status, place of residence, Education, Energy intake, breastfeeding, PA, social-economic, supplement, disease history, smoking, menopausal status, BMI, age, family history, alcohol
Niclis et al. (Argentina) (46)	A case-control study	317 BCs and 526 controls	FFQ (127items); 5 y prior	Dietary Inflammatory Index (DII)	Growing DII score enhanced the BC risk (OR:1.34; 95%CI:1.05-1.70)	Age, age at menarche, number of children, smoking, SES, family history, urbanization level, BMI,
Payandeh et al. (Iran) (53)	A case-control study	150 BCs and 150 controls, BCs with recently diagnosed (< 3 month)	FFQ (147items); 1 y prior, Daily intake	PDI, hPDI, uPDI	No association between none of the PDIs and the chance of BC in Iranian women.	BMI, PA, energy intake, education, marital status, breastfeeding, oral contraceptive, climacteric, alcohol, menarche, cigarette, supplements, comorbidities, HRT, menopause, weight (18 y), first pregnancy and family history, drug,
Foroozani et al. (Iran) (55)	A case-control study	1009 BCs and 1009 controls	FFQ (168items); Daily intake	Western dietary pattern (WD)	A positive and significant association was observed between higher adherence to a WD and risk of IDC (OR comparing highest with the lowest tertile: 2.45, 95%CI 1.88-3.17; p-trend < 0.001), whereas no significant association was observed between adherence to the WD and the risk of ILC (OR comparing highest with the lowest tertile: 1.63, 95%CI 0.63-3.25) (p for heterogeneity = 0.03).	Energy intake, menarche, garden stuff, breastfeeding, family history, smoking, chest X-ray, BBD, PA, first delivery, history of miscarriage, climacteric, OCP, BMI
Toorang et al. (Iran) (59)	A case-control study	477 BCs and 507 Controls(19-80 y)	FFQ (168items); 1 y prior	DASH Diet	DASH bring down the risk (OR for comparing extreme tertiles: 0.62; 95%CI 0.44-0.78; P_*trend*_ = 0.004).	Parity, Age, fertility treatment, energy intake, education, alcohol, smoking, PA, family history, marital status, OCP, BMI
Cao et al. (China) (9)	A case-control study	818 BCs and 935 controls	FFQ (149items);	Soy-Fruit-Vegetable Pattern	The pattern brings down the postmenopausal BC risk (4th vs. 1st quartile, OR: 0.57, 95%CI:0.41-0.80; P_*trend*_ < 0.001)	Age (diagnosis/enrollment/first full-term delivery), smoking, PA, oral contraceptives use, HRT, family history, history of BBD, menarche, number of full-term births, breastfeeding, height, energy intake, menopausal, education, BMI
Sasanfar et al. (Iran) (41)	A case-control study	412 BCs and 456 controls (19–80y) patients with recently diagnosed (< 1 year)	FFQ (168items); 1 y prior	Dietary inflammatory index (DII)	Individuals in the highest quartile of DII scores had 1.5 times higher odds of breast cancer than those with the lowest (OR:1.56, 95%CI:1.04–2.35, P_*trend*_ = 0.02).	Energy, educational level, parity, age, BMI, oral contraceptive use, tobacco use, alcohol use, marital status, PA, family history
Hosseini et al. (Iran) (17)	A case-control study	150 BCs and 150 controls, BCs with recently diagnosed (< 3 mo)	FFQ (147items);	Food Quality Score	A significant association between adherence to the FQS and odds of breast cancer in the fully adjusted model (OR: 0.58; P = 0.04) and in premenopausal women in the fully adjusted model (OR: 0.45; P = 0.02);	Menopause, Age, total energy, PA, education, marital status, SES, alcohol, smoking, vitamin medication, comorbidities, HRT, OCP, BP, menarche, weight (18y), number of children, breastfeeding ages, family history of BC, BMI, zinc, potassium, magnesium, calcium
Shamsi et al. (Pakistan) (32)	A case-control study	374 BCs and 750 controls. (18-75y)	FFQ	The Modified Alternate Healthy Eating Index-2010 (MAHEI-2010)	There was no association of BC with Alternate Healthy Eating Index 2010 score (OR: 1.85; 95%CI: 0.61–1.17; *p* = 0.291).	BMI, menopausal status, SES
Rigi et al. (Iran) (26)	A case-control study	350 BCs and 700 controls. (> 30y) BCs with recently diagnosed (< 6 month)	DS-FFQ (106 items); 1 y prior	Dietary glycemic index and glycemic load (GL)	Individuals in the highest tertile of dietary GI had 47% higher odds of BC than women in the lowest tertile (OR:1.47; 95%CI: 1.02–2.12). We found no significant association between dietary GL and odds of BC either before [OR: 1.35; 95%CI: 0.99–1.84)] or after adjustment for potential confounders (OR: 1.24; 95%CI: 0.86–1.79).	Alcohol, Marital, Age, energy intake, education, SES, place of residency, supplement use, family history of BC, PA, smoking, breastfeeding, menopausal, BMI
Harris et al. (US) (44)	Cohort study	45204 registered nurses and 1,477 BCs (27–44y) women)	HS-FFQ (124 items) and FFQ	An adolescent and early adulthood diet	Women in the fifth quintile of the inflammatory pattern score had multivariable adjusted HRs for premenopausal BC of 1.35 for adolescent diet (95%CI:1.06–1.73; P_*trend*_ 1/4 0.002) and 1.41 for early adulthood diet (95%CI: 1.11–1.78; P_*trend*_ 1/4 0.006) compared with women in the first quintile.	Age at first birth, height/weight/BMI (18y), age, total adolescence calories, menarche, adolescent PA, family history, parity, OCP, adult PA, alcohol, BBD, menopausal, hormone use
Kojima et al. (Japan) (54)	Cohort study	23,172 women and 119 BCs	FFQ (39 items);	Animal food pattern; Vegetable pattern; Dairy product pattern	The animal food pattern was significantly associated with a decreased risk of breast cancer morbidity among premenopausal women by HR 0.47 for the 2nd tertile (95%CI 0.22–1.00) and HR 0.42 for the 3rd tertile (95% CI:0.18–0.93), compared with the bottom tertile (p for trend = 0.04).	first birth, age, menarche, education, exogenous female hormones, duration, area, parity, family history, daily walking habits, alcohol, smoking, total daily energy intake, BMI,
Guinter et al. (US)(82)	Cohort study	261,959 woman and 1,968 BCs (35-74y)	FFQ (110items); 1 y prior	An Estrogen-Related Pattern	There is no relationship between ERDP and total, invasive, ER + /ER- of BC.	Family members with a history of BC (number), alcohol, total energy intake, BMI (baseline/30y), PHT, race, parity, menarche, hysterectomy, menopause, Age,
Haridass et al. (US) (21)	Cohort study	96,959 (California Teachers)	Semiquantitative FFQ (103items); 1 y prior	Paleolithic index: PALEO AHEI-2010 DASH aMED	In the analysis of women at risk of postmenopausal BC at baseline, higher AHEI-2010, aMED, and DASH scores were inversely associated with incident BC. Respectively, HRs (95%CIs) comparing quintile 5 to quintile 1 for AHEI-2010, aMED, and DASH indexes were 0.87 (0.78-0.97; P-trend = 0.004), 0.91 (0.82-1.02; P-trend = 0.03), and 0.89 (0.80-1.00; P-trend = 0.03).	Smoking, Age, HRT, family history, menarche, parity, OCP, SES, PA, total energy intake, alcohol, BMI, race,
Haraldsdottir et al. (Iceland) (83)	Cohort study	3,326 women and 97 BCs	FFQ and AGES-FFQ (107items);	Pattern1: Rye bread, cured meat/fish, viscera sausage, oat, Milk Pattern 2: Fish meal, Fish as a side, Fruit, Vegetables Pattern 3: Fish meal/oil, viscera sausage, oat, Milk, Pattern 4: Meat, Fish meal, Potatoes	Borderline inverse association was observed for the highest adherence to a pattern high in consumption of fish, blood/liver sausage, oatmeal, fish oil, and milk in adolescence (HR:0.6, 95%CI:0.4–1.0) (P_*trend*_ = 0.049)	Age at (entry/menarche, first child), BMI, education,
Petimar et al. (US) (31)	Cohort study	50,884 US women and 1,700 invasive BCs (mean follow-up, 7.6 y)	FFQ (110items); 1 y prior	DASH, AHEI-2010, AMED	Individuals in the highest quartile of DASH scores had a lower risk of invasive breast cancer compared with those in the lowest quartile (HR: 0.78; 95%CI: 0.67-0.90; P_–trend_ = 0.001), after excluding alcohol, AHEI-2010 was inversely associated with risk of ER–/PR– (HR:0.64; 95%CI: 0.42-0.98; P_–trend_ = 0.04) and ER–/PR–/HER2– subtypes.	Race, alcohol, education, recent mammogram, income, breastfeeding, BMI, HRT, height, OCP, PA, menopause, smoking, menarche, total energy intake, age at first live birth, family history, parity
Gardeazabal et al. (Spain) (47)	Cohort study	10,713 graduates and 100 confirmed and 168 probable incident BCs	FFQ (136items);	Dietary inflammatory index	The multivariable-adjusted HR for participants in the 4th quartile to the 1st quartile was 1.44 (95%CI:0.76-2.72; p_–trend_ = 0.339) when confirmed cases were analyzed, and 1.20 (95%CI 0.72-1.99; p_–trend_ = 0.757) for the probable cases. We neither observed statistically significant differences in regard to menopausal status.	The years from recruitment to the beginning of risk, Age, total energy intake, height, diabetes, university (years), HRT, relatives with a history of BC (number), months of breastfeeding, smoking, pregnancy (less than 30 years), PA, number of pregnancies of more than six months, alcohol, menopause, BMI, menarche,
Dela Cruz et al. (US) (33)	Cohort study	101,291 women in the MEC sample and 7,749 BCs	FFQ (180items); Daily intake	aMED; AHEI-2010; DASH; HEI-2015	The respective HRs for Q5 vs. Q1 were: 1.06 (95%CI: 0.98–1.14) for HEI-2015, 0.96 (95%CI:0.90–1.04) for AHEI-2010, 1.01 (95%CI: 0.94–1.09) for aMED, and 0.95 (95%CI: 0.88–1.02) for DASH (p_*trend*_ > 0.05 for all). No findings on the relationship between diet quality and BC risk.	Alcohol, Age, estrogen and progestin use, total energy intake, family history, BMI, menopause, smoking, parity, PA, age at first live birth, education, menarche,
Romanos-Nanclares et al. (Spain) (51)	Cohort study	10 812 women and 101 incident BCs	FFQ (136items); 1 y prior	Provegetarian dietary patterns (PVGs): a healthful PVG (hPVG); unhealthful PVG (uPVG)	A significant inverse association with BC (comparing tertile 2 vs. tertile 1, HR:0.55; 95%CI: 0.32-0.95) was identified for a modest overall PVG, but not for hPVG and uPVG separately.	HRT, height, menopause, family history, time since recruitment, smoking, total energy intake, PA, university(years), alcohol, months of breastfeeding, BMI, pregnancy (less than 30y), menarche, number of pregnancies of > 6 months,
Gardeazabal et al. (Spain) (39)	Cohort study	10713 young and middle-aged women and 100 definite cases and 168 probable BCs	FFQ (136items); Daily intake	Western pattern (WDP); Mediterranean pattern (MDP)	A higher adherence to a WDP was associated with an increased risk of overall BC (HR for confirmed BC Q4 v. Q1:1.70; 95%CI: 0.93- 3.12; P for trend = 0.045). Contrarily, adherence to a MDP was inversely associated with premenopausal BC (HR Q4 v. Q1;0.33; 95%CI: 0.12-0.91).	Menopause, Age, the time since recruitment, height, total energy intake, university (years), diabetes, relatives with a history of BC, smoking, HRT, PA, breastfeeding, alcohol, pregnancy (less than 30y), BMI, menarche, number of pregnancies of more than six months,
Park et al. (US) (43)	Cohort study	43563 participants and 2619 BCs	FFQ (110items); 1 y prior	D-OBS; E-DII	Whereas there was a suggestive inverse association for the highest vs lowest quartile of D-OBS (HR:0.92, 95%CI: 0.81-1.03). The highest quartile of E-DII was associated with risk of triple-negative BC (HR:1.53 95%CI: 0.99-2.35). When the two indices were combined, a proinflammatory/prooxidant diet (highest tertile of E-DII and lowest tertile of D-OBS) was associated with increased risk for all BC (HR: 1.13, 95%CI:1.00-1.27) and for triple-negative BC (1.72, 95%CI: 1.10-2.70), compared to an anti- inflammatory/antioxidant diet (lowest tertile of E-DII and highest tertile of D-OBS).	Alcohol, Race, degree of family history of BC, education, use of non-aspirin NSAIDs, BMI, hormone therapy, use of aspirin, menopausal, ever use of hormonal, the relationship between BMI and menopausal, recent mammogram screening, smoking, family history, years, PA,
Hidaka et al. (US) (15)	RCT	220 BCs; 440 controls	Diet History Questionnaire-I (DHQ-I) servings per day	Modern pattern; Traditional diet; Average pattern	Women with a Modern diet were more likely than women with a Traditional diet to develop ER- breast cancer: OR:3.33 (95%CI: 1.31-8.98); OR:3.12 (95% CI: 1.15-9.00). compared to an Average or Traditional diet, those with a Modern diet were more likely to develop an ER- tumor: OR: 2.56 (95%CI: 1.18-5.62); OR: 2.07 (95%CI: 0.89- 4.85).	Weight, Abnormal breast biopsy, menarche, Age at first birth, nulliparous, number of biopsies, race, Height, Age, family history
Jang et al. (Korea) (74)	Cohort study	511 BCs, recurrence includes a local (n = 12) or regional reappear (n = 16), heterolateral BC (n = 10), forane recurrence (n = 50) and 44 demises	A structured 24-h recall questionnaire daily intakes	Dietary Inflammatory Index	It was positively associated with the risk for cancer recurrence (HR:2.347, 95%CI: 1.17–4.71) and overall mortality (HR: 3.049, 95%CI: 1.08–8.83) after adjusting for confounding factors.	energy intake, time of life, AJCC grading, BMI, lymphatic metastasis, pausimenia, histological differentiation, Cancer size, treatment, subtype
Karavasiloglou et al. (US) (64)	Cohort study	110 were BC survivors	A 24-h dietary recall interview the past 24 h	The Healthy Eating Index (HEI) and the Mediterranean Diet Score (MDS)	Breast cancer survivors (HRHEI good vs. poor BC = 0.49, 95% CI: 0.25–0.97).	Alcohol, ethnicity, history of menopausal hormone therapy use, the time between diagnosis and completion of a questionnaire, daily energy intake, SES, self-reported prevalent chronic diseases at baseline, marital, smoking, BMI, PA
Wang et al. (China) (66)	Cohort study	3,450 BCs and 153 total deaths	FFQ (93items); 1 y prior	HEI-2015; Chinese Food Pagoda (CHFP)-2007, CHFP-2016; DASH	Participants in the highest quartiles of CHFP-2007, CHFP-2016 and DASH had 25-34% lower risk of total mortality (HR:0.66, 95%CI: 0.48-0.89 for CHFP-2007; HR:0.75, 95%CI: 0.55-1.01 for CHFP-2016; HR:0.66, 95%CI:0.49-0.91 for DASH), and 36-40% lower risk of breast cancer specific events (HR:0.64, 95%CI: 0.44-0.93 for CHFP-2007; HR:0.67, 95%CI: 0.45-0.99 for CHFP-2016; HR:0.60, 95%CI:0.40-0.90 for DASH) comparing to the lowest quartiles.	Immunotherapy (Age/BMI/PA), dietary survey, radiation, the interval between diagnosis and dietary survey, chemotherapy, total energy intake, comorbidity, education, TNM stage, income, HER2 status, marriage, menopausal status at diagnosis, PR status, ER status
Di Maso et al. (Italy) (68)	Cohort study	1453 women with BC	FFQ (78items); 2 years before diagnosis	Mediterranean Diet	Overall survival for 15 years improved (High:63.1%; low:53.6% p = 0.013).	Total energy intake, residence, ER/PR status, diagnosis (time/age), TNM stage, menopausal, education
Wang et al. (US) (14)	Cohort study	8,482 women with BC	FFQ at least 12 months after diagnosis date	Diabetes risk reduction diet (DRRD)	Women with higher post-diagnostic DRRD score had a 20% lower risk of breast cancer-specific mortality (top vs. bottom quintile HR:0.80; 95%CI:0.65-0.97; p-trend = 0.02) and 34% lower risk of all-cause mortality (HR:0.66; 95%CI:0.58-0.76; p-trend < 0.0001). Compared with women who consistently had lower score (≤ median) before and after diagnosis, those whose score improved from low to high had a lower risk of breast cancer-specific mortality (HR:0.77; 95%CI:0.62-0.95) and overall mortality (HR:0.85; 95%CI:0.74-0.97).	Change in BMI from pre- to post-diagnosis, post-diagnosis smoking status, (age/time) at diagnosis, (radiation, chemotherapy, and hormonal) therapy, (aspirin/alcohol/calories/SES/PA)post-diagnosis.ER status, menarche, disease stage, menopausal, pre-diagnosis BMI, parity, family history, menopausal hormone therapy use, BBD, OCP
Ergas et al. (US) (65)	Cohort study	3660 women diagnosed with invasive BC, 461 BC relapsers, and 655 demises	The Block 2005 Food Frequency Questionnaire (139items); daily intake	ACS; aMED; DASH; HEI-2015	Adjusted comparisons between extreme quintiles showed all 4 dietary quality indices to be inversely associated with all-cause mortality, suggesting a 21%-27% lower risk (ACS HR 1/4 0.73, 95%CI 1/4 0.56-0.95; aMED HR 1/4 0.79, 95%CI 1/4 0.61-1.03; DASH HR 1/4 0.76, 95%CI 1/4 0.58-1.00; HEI HR 1/4 0.77, 95%CI 1/4 0.60-1.01).	Hormonal therapies, race; radiation, education; chemotherapy, menopausal; type of surgery, PA; HER2, smoking; PR, cancer stage at diagnosis; ER, BMI
Lei et al. (China) (67)	Cohort study	1226 invasive early-stage BCs	FFQ (109items); 1 y prior	Western pattern; healthy pattern	Western eating pattern did not enhance the danger of BC relapse (Ptrend = 0.89), total death rate (Ptrend = 0.48) and BC-specific death (Ptrend = 0.75). There is no relationship between a healthy dietary pattern and prognosis	Total energy intake, Age, PA, comorbidities (number), BMI, diagnosis, menopausal, ER status, adjuvant hormonal therapy usage, PR status, radiotherapy, HER2 status, chemotherapy, histology,
Anyene et al. (US) (73)	Cohort study	3646 women diagnosed with BC	The Block 2005 FFQ (139items); the past 6 months	PDI; hPDI; uPDI	Increased concordance with hPDI was associated with a reduced hazard of all-cause (HR:0.93, 95%CI: 0.83–1.05) and non-breast-cancer mortality (HR:0.83, 95%CI: 0.71–0.98), whereas increased concordance with uPDI was associated with increased hazards (HR:1.07, 95%CI: 0.96–1.2 and HR:1.20, 95%CI:1.02–1.41, respectively).	total energy intake, PA, education, race, smoking, menopausal, HER2 status, diagnosis (age)
Chlebowski et al. (US) (69)	RCT	48,835 postmenopausal women	FFQ daily intake	Low-Fat Dietary Pattern	deaths after breast cancer (n = 134) were significantly reduced (40 deaths 0.025% per year v 94 deaths 0.038% per year; HR:0.65; 95%CI: 0.45-0.94; P = 0.02) by the dietary intervention. During the 16.1-year follow-up, with 3,030 incident breast cancers, deaths after breast cancer also were significantly reduced (234 deaths 0.085% per year v 443 deaths 0.11% per year; HR:0.82; 95%CI:0.70-0.96; *P* = 0.01) in the dietary group.	NO
Chlebowski et al. (US) (72)	RCT	48,835 postmenopausal women	FFQ daily intake	Low-Fat Dietary Pattern	Breast cancer overall survival was significantly greater for women in the dietary intervention group than in the usual-diet comparison group (10-year survival of 82% and 78%, respectively; HR: 0.78; 95%CI:0.65-0.94; *P* = 0.01). In the dietary group there were fewer deaths from breast cancer (68 vs. 120; HR:0.86; 95%CI,0.64-1.17), other cancers (36 vs 65; HR:0.76; 95%CI:0.50-1.17), and cardiovascular disease (27 vs. 64; HR:0.62; 95%CI, 0.39-0.99).	NO
Kim et al. (Korea) (11)	A cross-sectional study	232 BC survivors (stage I-III) (21-79 y)	Non-consecutive 3-day dietary record	Healthy pattern; Western pattern	higher Healthy dietary pattern scores tended to have lower dyspnea scores but higher insomnia scores. For dyspnea, the least squares mean was 8.86, 95%CI: 5.05–15.52 in the bottom quartile and 2.87, 1.62–5.08 in the top quartile (p for trend = 0.005).	PA (Age/BMI/disease stage/menopausal) at diagnosis, marital, education, time since surgery, energy intake
Lei et al. (China) (75)	A cross-sectional and longitudinal study	1462 BCs, (diagnosed within 1 year)	FFQ (109items); 1 y prior	Pattern1: vegetables and fruits pattern; Pattern2: grain and animal food pattern	Pattern2 reduced scores for role functioning (B,95%CI) (−0.744, −0.147 to −0.017), dyspnea (−0.092, −0.092 to −0.092) constipation (−1.355; −2.174 to −0.536). pattern1 were positively associated with scores for global health (B,95%CI) (1.282, 0.545-2.019), physical functioning (0.545, 0.037–1.053), emotional functioning (1.426; 0.653–2.200) cognitive functioning (0.822; 0.007–1.637), while inversely associated with scores for nausea and vomiting (−0.382, −0.694 to −0.071), dyspnea (−0.570, −0.570 to −0.570), insomnia (−1.412; −2.647 to −0.177), loss of appetite (0.722, −1.311 to −0.132), constipation (−2.028, −2.775 to −1.281) diarrhea (0.929; −1.481 to −0.377)	Factor scores of the other DP (Age/menopausal/BMI/PA/total energy intake/time-points) at follow-up, hormonal therapy, education, current usage of adjuvant, income, PR status, comorbidities, ER status, AJCC stage

FFQ is an abbreviation for food frequency questionnaire; y is an abbreviation for year; OR is an abbreviation for odd ratio; CI is an abbreviation for Confidence Interval; SQFFQ is an abbreviation for Semi-Quantitative Food Frequency Questionnaire; HER2 is an abbreviation for human epidermal growth factor receptor 2; HRT is an abbreviation for hormone replacement therapy; ER is an abbreviation for estrogen receptor; PR stands for progesterone receptor; CM is an abbreviation for Chinese medicine; DQX is an abbreviation for food frequency questionnaire; SES is the abbreviation of socioeconomic state; PA is the abbreviation of physical activity; BBD stands for Benign breast disease; OCP stands for oral contraceptive; QOL stands for the quality of life Score; AJCC is short for American Joint Committee on Cancer; DP stands for Dietary pattern; BMI stands for Body Mass Index; PHT is an abbreviation for postmenopausal hormone therapy; aMED is an abbreviation for alternate Mediterranean Diet; NSAIDs is an abbreviation for Non-steroidal Antiinflammatory Drugs; WHR is an abbreviation for Waist-to-Hip Ratio; HT is an abbreviation for hormone therapy; FFP is an abbreviation for parity and age at first full-term pregnancy; DASH is an abbreviation for Dietary Approaches to Stop Hypertension; PDI is an abbreviation for Plant diet index; hPDI is an abbreviation for healthy plant diet index; uPDI is an abbreviation for Unhealthy vegetal diet index; MIND is an abbreviation for Mediterranean-DASH Intervention for Neurodegenerative Delay; D-OBS is an abbreviation for dietary oxidative balance score; E-DII is an abbreviation for Energy adjusted-Dietary Inflammatory Index; ACS is an abbreviation for American Cancer Society; HEI-2015 is an abbreviation for Healthy eating index; IDC is an abbreviation for invasive ductal carcinoma; ILC is an abbreviation for invasive lobular carcinoma.

**TABLE 2:** Newcastle-Ottawa Scale of 41 studies in the systematic review.

Study	Selection				Comparability	Exposure			Total star
	**Definition of cases**	**Representativeness of cases**	**Selection of controls**	**Definition of controls**		**Assessment of outcome**	**Method of ascertainment**	**Non-response rate**	
Castelló et al. (37)	1	1	1	1	2	1	1	0	8
Sadeghi et al. (16)	1	1	1	1	2	1	1	0	8
Lu et al. (62)	1	1	1	0	2	1	1	1	8
Guinter et al. (63)	0	1	1	1	2	1	1	1	8
Krusinska et al. (22)	1	1	0	1	2	1	1	1	8
Krusinska et al. (23)	1	1	0	1	2	1	1	1	8
Jalali et al. (40)	1	1	1	1	2	1	1	1	9
Heidari et al. (18)	1	1	1	1	2	1	1	1	9
Shridhar et al. (52)	0	1	1	1	2	1	1	0	7
Sedaghat et al. (20)	1	1	1	1	2	1	1	1	9
Turati et al. (36)	1	1	1	1	2	1	1	1	9
Marzbani et al. (19)	1	1	1	1	2	1	1	1	9
Heidari et al. (58)	1	1	0	1	2	1	1	0	7
Stasiewicz et al. (25)	1	0	1	1	2	1	1	1	8
Hajji-Louati et al. (48)	0	1	1	1	2	1	1	1	8
Sasanfar et al. (49)	1	1	0	1	2	1	1	1	8
Aghamohammadi et al. (60)	1	1	0	1	2	1	1	1	8
Soltani et al. (57)	1	1	0	1	2	1	1	1	8
Cao et al. (34)	1	1	1	1	2	1	1	1	9
Cao et al. (80)	0	1	1	1	2	1	1	0	7
Cao et al. (35)	0	1	1	1	2	1	1	1	8
Hammad et al. (45)	0	1	0	1	2	1	1	0	6
Jacobs et al. (56)	0	0	1	1	2	1	1	1	7
Flores-García et al. (24)	1	1	1	1	2	1	1	1	9
Sheikhhossein et al. (81)	1	1	0	1	2	1	1	1	8
Rigi et al. (50)	1	1	0	1	2	1	1	1	8
Niclis et al. (46)	1	1	1	1	2	1	1	1	9
Payandeh et al. (53)	0	0	0	1	2	1	1	1	6
Foroozani et al. (55)	1	0	1	1	2	1	1	1	8
Toorang et al. (59)	1	1	0	1	2	1	1	0	7
Cao et al. (9)	0	1	1	1	2	1	1	1	8
Rigi et al. (26)	1	1	0	1	2	1	1	0	7
Sasanfar et al. (41)	0	1	0	1	2	1	1	1	7
Hosseini et al. (17)	1	1	0	1	2	1	1	1	8
Shamsi et al. (32)	1	1	0	0	2	1	1	0	6

**TABLE 3:** Newcastle-Ottawa Scale of 20 studies in the systematic review.

Cohort study	Selection				Comparability	Outcome			Total star
	**Exposed cohort**	**Non-exposed cohort**	**Ascertainment of exposure**	**Outcome of interest**		**Assessment of outcome**	**Length of follow-up**	**Adequacy of follow-up**	
Harris et al. (44)	0	1	1	1	2	1	1	1	8
Kojima et al. (54)	1	1	1	1	2	1	1	0	8
Guinter et al. (82)	1	1	1	1	2	1	1	1	9
Haridass et al. (21)	0	1	1	1	2	1	1	0	7
Haraldsdottir et al. (83)	1	1	1	1	2	1	1	0	8
Petimar et al. (31)	1	1	1	1	2	1	1	0	8
Gardeazabal et al. (47)	0	1	1	1	2	1	1	1	8
Dela Cruz et al. (33)	1	1	1	1	2	1	1	1	9
Romanos-Nanclares et al. (51)	0	1	1	1	2	1	1	1	8
Gardeazabal et al. (39)	0	1	1	1	2	1	1	1	8
Park et al. (43)	1	1	1	1	2	1	1	0	8
Jang et al. (74)	1	1	1	1	2	1	1	1	9
Karavasiloglou et al. (64)	1	1	1	0	2	1	1	1	8
Wang et al. (66)	1	1	1	0	2	1	1	1	8
Di Maso et al. (68)	1	1	1	0	2	1	1	1	8
Wang et al. (14)	0	1	1	0	2	1	1	1	7
Ergas et al. (65)	1	1	1	0	2	1	0	1	7
Lei et al. (67)	1	1	1	0	2	1	1	1	8
Anyene et al. (73)	1	1	1	0	2	1	1	1	8

**TABLE 4:** JBI Scale of 2 studies in the systematic review.

Author	A	B	C	D	E	F	G	H	I	J	Total score
Kim et al. (11)	2	1	2	2	1	2	2	2	2	2	18
Lei et al. (75)	2	1	2	2	2	2	2	2	2	2	19

A: Whether the research objectives of the study are clear, Whether the basis for the establishment of the topic is sufficient; B: How the population was selected (whether the study subjects were randomly selected, whether stratified sampling was taken to improve sample representativeness); C: Whether the inclusion and exclusion criteria of the sample are clearly described; D: whether clearly describes the sample characteristics; E: Whether the tools for data collection are reliable and valid (e.g., investigator surveys, how reproducible are the findings); F: What are the measures to verify the authenticity of the information; G: Whether ethical issues are taken into account; H: Whether the statistical method is correct; I: Whether the statement of the findings of the study is appropriate and accurate (whether the results and inferences are distinguished, and whether the results are faithful to the data rather than inferences); J: does it make a clear elaborate of the value of the study. 0 points: does not meet the requirements; 1 point: mentioned but not described in detail; 2 points: detailed, comprehensive, correct description.

### Dietary patterns and the risk of BC

The relationship between dietary patterns and the risk of BC has been studied by researchers in 16 different countries, particularly in relation to healthy, Mediterranean, inflammatory, plant-based, and Western dietary patterns (Supplementary material).

Healthy eating patterns were investigated in populations from various geographical locations, including, Iran (*n* = 5), the United States (*n* = 3), Pakistan (*n* = 1), Poland (*n* = 2), and Mexico (*n* = 1). This pattern reduced the risk of BC, whereas unhealthy eating patterns increased the risk (16–24). An unhealthy diet was positively related to the occurrence of postmenopausal BC through its proinflammatory potential. In contrast, regular consumption of low-processed vegetable products and fish was negatively related to the occurrence of cancer (25). Another study in Iran found a significant positive correlation between dietary glycemic index and the incidence rate of BC (26), while high dietary fiber intake, such as beans and grains, was shown to reduce the risk of estrogen receptor negative (ER–) and progesterone receptor negative (PR–) BC in the United States (27). Meat and processed meat diets were associated with a higher risk of BC in a Chinese study (28), while the consumption of vegetables, fruit, and soybeans reduced the risk of postmenopausal BC, especially ER– and ER–/PR– subtypes (9). The consumption of fresh fruit and nuts was negatively correlated with the risk of menopausal BC, and foods with a high sodium content were positively correlated with the risk of menopausal BC in a South African study (29), and a multigrain diet reduced the risk of BC in a South Korean study (30). These findings were consistent with the results of the study on healthy eating patterns (16, 18). However, a study conducted by American researchers showed that the Alternative Healthy Eating Index–2010 (AHEI-2010) had a weak (but insignificant) correlation with the risk of BC, but after excluding alcohol, it was negatively correlated with the risk of ER–/PR– and ER–/PR–/human epidermal growth factor receptor 2 (HER2–) BC (31), and there was no relationship between this index and BC risk in another study conducted in Pakistan (18, 19, 32, 33).

Studies on the effects of a Mediterranean diet, characterized by high intakes of fish, vegetables, beans, boiled potatoes, fruit, olives and vegetable oils, and a low intake of fruit juice, were carried out in the United States (*n* = 3), China (*n* = 2), Spain (*n* = 1), and Italy and Switzerland (*n* = 1), while studies of ‘prudent’ dietary patterns similar to a Mediterranean-style diet (34) have been carried out in China (*n* = 1), Spain (*n* = 1), and Poland (*n* = 2). A higher score for a Mediterranean diet was negatively related to BC in some studies (21–23, 28, 35, 36), especially after the menopause (37–39), while the Spanish study and two studies in the United States showed only a weak or no correlation (31, 33, 39). Prudent dietary patterns were associated with a lower risk of BC in one study (34), but had no observable effect on BC in the Spanish study (37–39).

The effects of an inflammatory diet were investigated in Iran (*n* = 2), the United States (*n* = 2), Spain (*n* = 1), Poland (*n* = 1), Jordan (*n* = 1), France (*n* = 1), and Argentina (*n* = 1). Inflammatory dietary patterns, including high intakes of sugary soft drinks, refined grains, red and processed meat, margarine and other hydrogenated fats, and low intakes of green leafy vegetables, cruciferous vegetables, coffee, increased the risk of BC in premenopausal and overweight postmenopausal women (40–44). In addition, a low dietary inflammation index reduced the risk of BC in obese women (45, 46). However, there was no significant relationship between the dietary inflammation index and the incidence rate of BC in a Spanish study (47), while a French study found that an inflammatory diet only increased the risks of ER+, PR+, or HER2+ breast tumor subtypes, but found no relationship with triple-negative (ER–, PR–, and HER2–) BC (48).

Plant-based diets have been investigated in Iran (*n* = 3), China (*n* = 1), Spain (*n* = 1), and North India (*n* = 1). The plant diet index (PDI) and a healthy PDI were negatively correlated with the incidence rate of BC (28, 49–51), while an unhealthy PDI was associated with an increased risk (50). Lacto-ovo vegetarians (whose diet includes plants, dairy products, and eggs) had a lower risk of BC compared with meat eaters and lacto vegetarians (vegetarian diet and dairy products) according to a multicenter case-control study of women in northern India (52). However, there was no significant correlation between PDI and the incidence of BC in the Iranian study and another study in Japan (53, 54).

The effects of a Western dietary pattern were investigated in Iran (*n* = 1), Spain (*n* = 2), and Mexico (*n* = 1). This pattern (high intakes of fat, sugar products, and red and processed meat) increased women’s risk of BC in some studies (24, 29, 37–39). However, some studies found a positive correlation between a Western diet and the risk of invasive ductal carcinoma of the breast, but no significant correlation with the risk of invasive lobular carcinoma (55, 56).

Four beneficial dietary patterns are summarized: a healthy diet, Mediterranean diet, anti-inflammatory diet, and plant-based diet. Other dietary patterns negatively related to the risk of BC include dietary approaches to stop hypertension (DASH) (31, 57–59), Mediterranean-DASH Intervention for Neurodegenerative Delay (MIND) (60), a diet with a high intake of vitamins, trace elements, carbohydrates, fiber, and protein (61), and traditional diets (15, 56, 62). Dietary patterns positively related to the risk of BC include an estrogen-related dietary pattern (63) and a modern diet (15).

### Dietary patterns and prognosis of BC

The relationship between dietary patterns and BC prognosis has been studied in four countries, particularly focusing on healthy, Mediterranean, and DASH diets. Two studies on healthy eating patterns conducted in the United States found that this pattern had the potential to reduce patient mortality (64, 65), while two studies in China found no such relationship (66, 67).

Two studies in the United States and one in Italy investigated the effects of a Mediterranean diet. The third national health and nutrition examination survey in the United States conducted in 2019 found no correlation (64), while the survey in 2021 showed that this diet was negatively related to BC mortality (65). In the Italian study, the 15-year overall survival rate among patients with high compliance to a Mediterranean diet was better than that among patients with low compliance, but there was no significant correlation with either increased or decreased mortality rates (68).

One study of the DASH diet in the United States showed that this diet reduced mortality in BC patients (65); however, a Chinese study found that adherence to the DASH diet was associated with higher mortality compared with adherence to the Chinese food pagoda CHFP-2007/2016 (66).

Dietary patterns shown to reduce mortality and improve overall survival among patients with BC include a low-fat diet (13, 69–72), diabetes risk-reduction diet (14), plant-based diet (73), and anti-inflammatory diet (74). However, different studies have shown different results in terms of all-cause mortality, specific mortality, total mortality, cancer recurrence, and non-BC-related deaths among BC patients, indicating the need for more research.

### Dietary patterns and quality of life in BC

Two studies investigated the effects of dietary patterns on quality of life in patients with BC. A Korean study showed that healthy eating habits improved dyspnea but increased insomnia in specific populations (11). A Chinese study investigated the relationship between eating more grain and animal products and poorer functions, including respiratory function and constipation, and the effects of a high-fruit and vegetable diet in improving quality of life, including physical, emotional, and cognitive functions, as well as reducing common gastrointestinal reactions, breathing problems, and insomnia (75).

## Discussion

A search was conducted to find and analyze recent studies examining the influence of dietary patterns on BC, to identify dietary patterns likely to prevent BC and improve its prognosis, and enhance the quality of life for BC survivors. The available data suggested that healthy dietary patterns had the most scientific evidence to support their beneficial effects compared with other dietary patterns. The different dietary patterns are discussed below in order of scientific evidence.

Adhering to a healthy diet pattern reduced the risk of BC, BC recurrence, all-cause mortality, and overall mortality, and improved the quality of life (especially in postmenopausal women and hormone receptor-negative women). This dietary pattern was characterized by low intakes of carbohydrates, red and processed meats, and sweet foods, and increased intakes of protein, folic acid, calcium, vitamin D, and fiber. Thus, even though physical activity decreased, the dietary fat energy percentage also decreased and body weight remained unchanged. This was consistent with a study of low-fat diet patterns (10). The results of the study on a prudent diet pattern (23), characterized by more frequent consumption of dairy products, fruit, vegetables, wholewheat bread, fish, and fruit juice, were similar, especially in premenopausal women, with significance for hormone receptor-positive and -negative tumors. This dietary pattern may reduce the risk of BC by regulating plasma lipid biomarkers, and improve the prognosis by reducing the overexpression of RhoA and Rho-associated protein kinase-related (8, 34).

Current evidence shows that high adherence to a Mediterranean diet significantly reduces the incidence rate of BC, especially invasive ductal and lobular BC, it is more significant for ER- or ER+, has the best anti-tumor-metastasis effect, and reduces disease recurrence, overall mortality, and other complications such as cardiovascular disease, and has a greater beneficial impact than a prudent dietary pattern (37, 68). The mechanism involves reducing glucose, weight, and waist circumference, improving biochemical parameters, reducing the biological activities of insulin-like growth factor 1 (IGF-1), testosterone, and estradiol, increasing antioxidation, and repairing DNA (36, 65). A summary analysis of the individual components of the Mediterranean diet showed that the protective effect was mainly attributable to fruit, vegetables, and whole grains (21, 35). The protective effect of the Mediterranean diet, which contains fish, beans, nuts, seeds, whole grains, and vegetables, may be due to specific chemical components, such as lignans and polyphenols, or to its wider nutrient components, such as fatty acids, resveratrol, organic sulfur compounds, quercetin, kaempferol, and apigenin, as well as the common micronutrients zinc and selenium, and phytochemicals, such as flavonoids, carotenoids, vitamins C and E, vitamin A, natural retinoids, and omega-3 polyunsaturated fatty acids. An increase in circulating tumor cells in the body was shown to be delayed by low-fat components (76). These compounds have demonstrated anticancer properties including affecting the growth and progression of BC, cancer cell cycle growth arrest, apoptosis, inflammation, angiogenesis, and DNA methylation of the gene, which can prevent the progress of obesity-related BC, and has a positive impact on all-cause mortality (77, 78).

Mediterranean-DASH Intervention for Neurodegenerative Delay (MIND), a Mediterranean diet, and DASH diet are all plant-based diets, emphasizing the consumption of fruits and green leafy vegetables, beans, whole grains, nuts, fish, and poultry, and low intakes of saturated fats and red meat. These diets are sources of carotenoids, flavonoids, folic acid, and vitamin E. The mechanisms of this type of diet reduce the risk and mortality of BC similar to the effects of a Mediterranean diet (21, 66).

Compliance with a plant-based diet reduces the risk of BC, especially those types of BC that are more likely to become invasive, and improves the overall survival rate, especially in patients with ER–, HER2 basal-like, and luminal A BC. This diet includes more fruit and vegetables, especially cruciferous and yellow/orange vegetables, beans, nuts, seeds, and whole grains (12). The mechanism involves the reduction of IGF-1, blood glucose, and total cholesterol, while phytochemicals (allicin, hesperidin, and astragalus polysaccharide) included in this diet significantly inhibit the growth of primary tumors and metastatic lesions by reducing the expression of genes (50). Although there is a negative correlation trend between soluble fiber and estradiol levels, serum estradiol and estrone levels are not related to dietary fiber. A plant-based diet can thus improve the prognosis of BC by affecting the intestinal microbiota and hormone levels (21, 66); however, further studies are needed to clarify this.

In addition, a low-glucose diet, characterized by the intake of glucose equal to or lower than the average fasting level, improved insulin resistance (HOMA-IR) and other cancer-related serum biomarkers in some studies, thereby favorably regulating postmenopausal obesity as a postmenopausal BC prevention strategy (79). Other dietary patterns that improve the prognosis and quality of life of BC patients, such as an anti-inflammatory diet, have been shown to improve the prognosis of BC patients by reducing cardiovascular mortality (25).

It is also important to understand the mechanisms of dietary patterns that are negatively associated with disease, such as a Western diet, which is characterized by higher intakes of red and processed meat, dairy products, and saturated fats. A Western diet can lead to BC via the production of several carcinogenic compounds associated with cooking and processing meat, including nitrates, nitrites, heterocyclic amines, and polycyclic aromatic hydrocarbons (55). In an inflammatory diet, inflammatory markers increase BC risk by stimulating angiogenesis, cell proliferation, and migration, and preventing apoptosis, while other inflammatory biomarkers may reduce quality of life. In addition, the key mediators of the inflammatory response promote tumor growth, angiogenesis, and invasion through the influence of insulin resistance and increased cytokines (25). However, results on this topic are currently lacking, and more correlation studies are needed.

This study showed that a balanced dietary pattern [large amounts of protein (mainly white meat), fruits, and vegetables (rich in vitamins and minerals), nuts, beans, low omega-3 fatty acid diet of fish and seafood, whole grains, vegetable oil, and low intake of spices] may prevent BC and improve BC prognosis. However, except for alcohol intake, no studies have yet demonstrated a consistent and significant correlation for any specific foods, and the study of dietary patterns is affected by regional and cultural backgrounds. The beneficial dietary patterns summarized in this review should thus be interpreted carefully in view of the exploratory nature of the analysis. The findings are inconsistent, indicating the need for further studies to explore this topic.

Niclis et al.’s case-control study of inflammatory dietary patterns showed an association with disease risk, whereas Gardeazabal et al.’s cohort study showed no such association, which may reflect recall bias rather than a true difference (46, 47). Some studies showed that inflammatory diets increased the risk of BC (ER+, PR+, HER2+), but few studies have examined hormone-negative or triple-negative BC, and the effect of diet on heterogeneous breast risk or prognosis remains unclear (25). Foroozani et al.’s study did not assess the role of dietary patterns based on the histological subtype of breast cancer (55). Finally, although most of the included studies adjusted for a large number of confounding factors (body mass index, family history, smoking, etc.) that may confuse the association between dietary patterns and BC, not all studies adjusted for all potential confounding factors, such as physical activity and smoking. Future research should thus pay attention to this aspect. In addition, more evidence is required regarding prior and posterior eating patterns, study area, menopausal status, and hormonal status, to produce more conclusive results.

### Limitations

This study had some limitations. We only retrieved published literature, which may have led to publication bias due to incomplete literature collection. In addition, the reproducibility of dietary patterns was poor, due to differences in dietary research methods, evaluation methods (factor analysis, reduced rank regression), research populations, and regions. Because of the high heterogeneity among the included studies, the results were not analyzed by objective quantitative methods, and we were therefore unable to perform subgroup analyses due to the limited number of included studies.

Notably, despite the large number of studies, nutritional studies often produced inaccurate and/or contradictory results. In addition, BC is a multifactorial disease, and diet is only one of numerous risk factors associated with its pathology.

In addition, nutrition research has some problems. First, food surveys do not conform to reality, and different patients have different reactions to the same food as a result of interactions among genes, nutrients, and the intestinal microbiota. In addition, food nutritional profiles are affected by food practices and storage (e.g., fresh vegetables are chemically different from processed vegetables). Although clinical trials can be used to investigate simple and short-term problems, they are unsuitable for studying long-term diseases: it is difficult to randomly assign different diets to different populations and track them for many years to determine if a certain food is related to specific diseases. Furthermore the confounding factors in observational studies were not controlled, potentially leading to inaccurate results.

## Conclusion

Despite these limitations, the results of different types of studies (with different environments, methods, and patients) suggested similar conclusions, indicating a link between dietary patterns and clear health outcomes. Based on these findings, it is better to propose a “healthy” diet model, rather than claim any impact of specific foods or food ingredients. BC patients should be encouraged to improve their dietary habits before, during, and after treatment, in order to improve their long-term survival and quality of life.

This study systematically reviewed the impact of dietary patterns on BC risk, treatment outcomes, prognosis, and quality of life. On one hand, most studied dietary patterns tended to prevent the occurrence of BC, while fewer studies examined their effects on the prognosis and quality of life of survivors. On the other hand, more RCTs are needed to demonstrate the effects of these dietary patterns on cancer-specific outcomes (event-free survival, recurrence), and more research is required to clarify the mechanisms underlying the correlation of dietary patterns with BC based on biological processes.

## Data availability statement

The original contributions presented in this study are included in the article/Supplementary material, further inquiries can be directed to the corresponding author.

## Author contributions

YB and JZ: conceptualization, methodology, formal analysis, and writing—original draft. HS and MX: investigation. JCQ and SJ: resources. YL and JLQ: writing—review and editing. All authors contributed to the article and approved the submitted version.
